# Computational protocol to identify shared transcriptional risks and mutually beneficial compounds between diseases

**DOI:** 10.1016/j.xpro.2024.102883

**Published:** 2024-02-12

**Authors:** Hua Gao, Mao Zhang, Richard A. Baylis, Fudi Wang, Johan L.M. Björkegren, Jason J. Kovacic, Arno Ruusalepp, Nicholas J. Leeper

**Affiliations:** 1 Department of Surgery, Division of Vascular Surgery, Stanford University School of Medicine, Stanford, CA 94305, USA; 2 Stanford Cardiovascular Institute, Stanford, CA 94305, USA; 3 Department of Medicine, Division of Cardiovascular Medicine, Stanford University School of Medicine, Stanford, CA 94305, USA; 4 Department of Medicine, Division of Cardiology, University of California, San Francisco, San Francisco, CA 94143, USA; 5 Department of Medicine, Karolinska Institute, Huddinge, Sweden; 6 Department of Genetics and Genomic Sciences, Institute of Genomics and Multiscale Biology, Icahn School of Medicine at Mount Sinai, New York, NY 10029, USA; 7 Cardiovascular Research Institute, Icahn School of Medicine at Mount Sinai, One Gustave L. Levy Place, New York, NY 10029, USA; 8 Victor Chang Cardiac Research Institute, Darlinghurst, NSW, Australia; 9 St. Vincent’s Clinical School, University of NSW, Sydney, NSW, Australia; 10 Department of Cardiac Surgery and The Heart Clinic, Tartu University Hospital and Department of Cardiology, Institute of Clinical Medicine, Tartu University, Tartu, Estonia

**Keywords:** Bioinformatics, Cancer, Health Sciences, RNAseq

## Abstract

The accumulation of omics and biobank resources allows for a genome-wide understanding of the shared pathologic mechanisms between diseases and for strategies to identify drugs that could be repurposed as novel treatments. Here, we present a computational protocol, implemented as a Snakemake workflow, to identify shared transcriptional processes and screen compounds that could result in mutual benefit. This protocol also includes a description of a pharmacovigilance study designed to validate the effect of compounds using electronic health records.

For complete details on the use and execution of this protocol, please refer to Gao et al.^1^ and Baylis et al.^2^

## Highlights

• This protocol identifies shared transcriptional patterns between diseases
• Accommodates novel, user-supplied datasets for enhanced generalizability
• Leverages drug perturbation data to screen for mutually beneficial drugs
• Validates putative drugs using data from electronic medical records

## Abstract (editor highlights)

Publisher’s note: Undertaking any experimental protocol requires adherence to local institutional guidelines for laboratory safety and ethics.

## Graphical abstract

**Figure:**
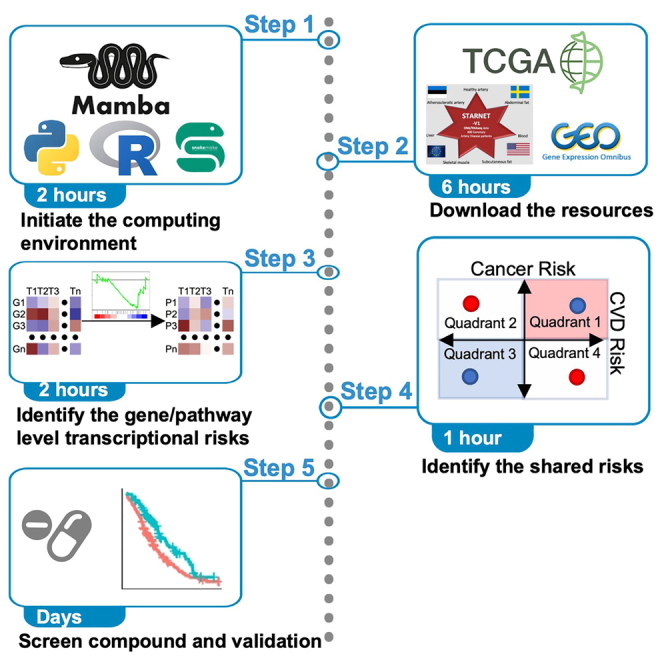

## Before you begin

The protocol below describes the specific steps used to identify the shared transcriptional risks between cancer and atherosclerosis using The Cancer Genome Atlas (TCGA) datasets for the various cancer subtypes and the Stockholm-Tartu Atherosclerosis Reverse Network Engineering Task (STARNET) and Biobank of Karolinska Endarterectomy (BiKE) atherosclerosis datasets. However, the protocol is adaptable to any analogous datasets from which summary statistics of transcriptional risks can be derived.

This section includes recommendations for the hardware specifications, and instructions for the pipeline localization, the computing environment setup, and some restricted resources downloading.

### Institutional permissions

This protocol involves pharmacovigilance study design that leverages de-identified electronic health records. While the code is initially tailored for use on the Stanford STARR platform and the necessary permissions required, it is important to note that any warehouse adhering to the Observational Medical Outcomes Partnership (OMOP) Common Data Model (CDM) would be compatible with this protocol.

### Hardware

Most of the protocol steps can be executed on a modern personal computer, such as one equipped with an Intel Core i7 processor and 16 GB of memory. However, for the drug screening step, it is highly recommended to utilize a high-performance computing cluster. Doing so will significantly reduce the processing time, condensing it from weeks to just a few days. Additionally, this pipeline is primarily designed for execution on a Linux system.

### Downloading the pipeline

**Timing: <10 min**

1. Within a working directory, retrieve the pipeline code from https://github.com/ghbore/protocol-cancer-cvd-similarity (https://doi.org/10.5281/zenodo.10493942) by downloading the zip archive and then extracting it, or by using the git-clone command:

> git clone https://github.com/ghbore/protocol-cancer-cvd-similarity.git

### Installing the computing environment

**Timing: 2 h**

2. To facilitate setting up the computing environment, a Conda environment YAML file is included along with the pipeline code. Thus, before the environment initiation, it is essential to have Conda or Mamba installed on the system. Otherwise, refer to the “Mamba Installation Guidelines” (https://github.com/conda-forge/miniforge) for detailed instructions.
3. Create a conda virtual environment and activate that environment.

> cd protocol-cancer-cdv-similarity

> mamba create --name protocol --file workflow/envs/env.yaml

> conda activate protocol

4. Install dependent R packages.

> bash workflow/envs/post.sh

### Downloading the restricted resource

**Timing: 1 h**

5. Given that the CVD BiKE dataset is array-based expression profiles using Affymetrix Human Genome U133 Plus 2.0 Array, and the official array annotation file is under restricted access, researchers can download the annotation file following the instruction in this Thermofisher webpage, and then move the downloaded file to “resources/bike/HG-U133_Plus_2-na36-annot-csv.zip”.
6. Regarding the STARNET CVD dataset, researchers with authorized access can download the raw RNA-Seq data and phenotype data from dbGaP: phs001203.v3.p1, re-analyze them to generate the summary statistics, and copy the summary statistics to the “resources/starnet/” directory as demonstrated below. Alternatively, the original summary statistics of the STARNET dataset are available upon request from Prof. Björkegren. Researchers can also skip the gene-level analysis and start from the pathway-level statistics provided in the “all resources and results bundle” as indicated in the “key resources table”.

## Key resources table

**Table:** 

REAGENT or RESOURCE	SOURCE	IDENTIFIER
**Deposited data**		

TCGA dataset: RNA-Seq	GDC Pan-Cancer	HTSeq-FPKM-UQ
TCGA dataset: basic phenotype	GDC Pan-Cancer	Basic phenotype
TCGA dataset: clinical information	GDC TCGAbiolinks	Bioconductor/TCGAbiolinks
BiKE dataset: expression array	NCBI GEO	GSE21545
BiKE dataset: array annotation	Thermo Fisher Scientific	webpage
STARNET summary statistics	Franzén et al.^3^	dbGaP phs001203.v3.p1
OCTAD compiled LINCS database	Zeng et al.^4^	octad.db_0.99.0
GENCODE annotation (human v36)	GENCODE	GENCODE Release 36
All resources and results bundle	This paper	https://doi.org/10.5281/zenodo.10032149

**Software and algorithms**		

Snakemake (>= 7.0.0)	N/A	github.com/snakemake/snakemake
R (>= 4.1.0)	CRAN	cran.r-project.org
R clusterProfiler package	Wu et al.^5^	Bioconductor/clusterProfiler
R Seurat package	Hao et al.^6^	github.com/satijalab/seurat

## Step-by-step method details

### Workflow configuration

**Timing: 30 min**

This pipeline is encapsulated in a Snakemake workflow, making it highly accessible and user-friendly. The user experience is further enhanced by the centralized configuration options housed in the “config/config.yaml” file. These configurations include.

• Gencode Annotation version. Choose the preferred Gencode annotation version from the available options listed here: https://www.gencodegenes.org/human/releases.html. The default value is “36”, in order to replicate the published results.
• The database used for pathway enrichment analysis. The options include MSigDB hallmark database, KEGG pathway, GO molecular function terms, GO biological process terms, or even a custom term-gene mapping file in the TSV format. The default is “hallmark”.

# Available database for enrichment analysis:

# 1. hallmark

# 2. KEGG

# 3. GO_MF

# 4. GO_BP

# 5. Custom pathay term-gene mapping file in the TSV format

#  with at least two columns,

#  one for the pathway term (ID and/or description),

#  and one for the gene (ensembl, entrez, and/or symbol).

#  For example:

#  ID  entrez  description

#  hsa01100 10   Metabolic pathways

#  hsa01100 100  Metabolic pathways

enrichment_analysis_database: hallmark

# if hallmark DB is chosen, use this version

msigdb_version: "7.2"

• Custom dataset registration, allowing researchers to integrate their custom datasets into the analysis. The configuration includes,
  a. Dataset name. Define unique dataset names for easy identification and reference.
  b. Gene level risk statistics file. The file can be a TSV file, or Excel file, and must contain the following minimal columns:
    i. Gene IDs, which can be ensembl (Ensembl gene ID), entrez (NCBI Entrez gene ID), or symbol (gene symbol),
    ii. A beta value, the effect size, such as survival log hazard ratio and correlation coefficient,
    iii. A pval value, the statistical *P*-value associated with the corresponding “beta” value.

# Custom dataset registration.

datasets:

 custom_1: # Dataset name

  # Gene-level risk profile file.

  # The file can be a TSV or Excel, at least three columns:

  # 1. gene ID, such as ensemble, entrez, symbol

  # 2. beta, the effect size

  # 3. pval, the statistical P-value

  gene: "path/to/gene_level_risk_profile"

 AOR_vs_MAM: # An example

  gene: "custom/AOR_vs_MAM_DGElist.xlsx"

• P-value and effect size cutoffs to remove noise and outlier genes.
• Dataset clustering configuration, further including,
  a. Analysis name. Define unique names for different analyses combining various datasets and resolution setting. Each name will serve as a prefix for corresponding output filenames (e.g., “reports/cancer_only-clustering.html” for analysis “cancer_only”).
  b. Associated datasets. Specify the list of datasets included in each analysis.
  c. Resolution parameter. This parameter controls the granularity of clustering. Higher values (above 1.0) lead to a larger number of communities, while lower values (below 1.0) result in a smaller number of communities. The optimal value depends on the specific goals. Additional details are available here: https://satijalab.org/seurat/reference/findclusters.

# Clustering parameters.

dataset_clustering:

 cancer_only: # Analysis name

  datasets: # The list of datasets included in the analysis

  [

   "ACC", "BLCA", "BRCA", "CESC", "CHOL", "COAD",

   "ESCA", "GBM", "HNSC", "KIRC", "KIRP", "LAML", "LGG",

   "LIHC", "LUAD", "LUSC", "MESO", "OV", "PAAD", "READ",

   "SARC", "SKCM", "STAD", "THCA", "UCEC", "UCS", "UVM"

  ]

  resolution: 1

• Dataset grouping configuration. The grouping configuration may depend on dataset clustering results. The configuration further includes:
  a. Analysis name. Similar to clustering, specify distinct names for various grouping configurations. These names will again prefix output filenames, for example, “reports/cancer_clusters-shared_risks.html” for the analysis “cancer_clusters”.
  b. Group names and their associated datasets. Assign meaningful names to each cluster of datasets.

# Dataset group definition.

dataset_grouping:

 athero_vs_cancer: # Analysis name

  atherosclerosis: # List of datasets in this cluster

   ["AOR_vs_MAM", "AOR_duke", "AOR_syntax", "bike_plaque"]

  cancer:

  ["ACC", "BLCA", "BRCA", "CESC", "CHOL", "COAD",

   "ESCA", "GBM", "HNSC", "KIRC", "KIRP", "LAML", "LGG",

   "LIHC", "LUAD", "LUSC", "MESO", "OV", "PAAD", "READ",

   "SARC", "SKCM", "STAD", "THCA", "UCEC", "UCS", "UVM"

  ]

### Resource downloading and compiling

**Timing: 5 h**

This step focuses on localizing and compiling essential resources, including the TCGA datasets, the BiKE dataset (except for the array annotation file, see “before you begin” step 5), GENCODE annotation files, GSEA hallmark gene sets, and the OCTAD-compiled LINCS database.

1. Download the dependent resources.

> snakemake --cores all Download

>

> tree resources

# resources/

# ├── bike

# │ ├── HG-U133_Plus_2-na36-annot-csv.zip

# │ └── series.gz

# ├── gencode.gff.gz

# ├── gencode.metadata.entrez

# ├── gsea

# │ └── hallmark

# ├── lincs

# │ ├── GPL20573.tsv.gz

# │ └── octad.db_0.99.0.tar.gz

# ├── starnet

# │ ├── AOR_vs_MAM_DGElist.xlsm

# │ └── AORsyntax_duke_Cor_P.xlsm

# └── tcga

# ├── all_indexed_clinical.rda

# ├── basic_pheno.tsv.gz

# └── fpkm-uq.tsv.gz

***Note:*** All steps should be triggered within the pipeline directory “protocol-cancer-cdv-similarity”. Ensure that you remain within the designated computing environment. Be prepared to provide the file “resources/bike/HG-U133_Plus_2-na36-annot-csv.zip” manually in advance, as detailed in "Before you begin" step 5. Similarly, ensure that the files inside “resources/starnet/” have been prepared in advance, as detailed in "Before you begin" step 6.

***Note:*** Specifically, the latest TCGA clinical data can be downloaded through Bioconductor/TCGAbiolinks package, as showcased in the script “workflow/scripts/tcga-clinical.R”. Briefly, for each TCGA project such as TCGA-BRCA, the “GDCquery_clinic” function retrieves a 158-column table of clinical information. These tables were then merged and saved to “resources/tcga/all_indexed_clinical.rds” for further extraction.

2. Compile the resources.

> snakemake --cores all Compile

>

> tree resources

# resources/

# ├── bike

# │ ├── anno.rds

# │ └── data.rds

# ├── gencode.rds

# ├── lincs

# │ ├── landmark.rds

# │ └── lincs.rds

# ├── starnet

# │ ├── AOR_vs_MAM.rds

# │ ├── AOR_syntax.rds

# │ └── AOR_duke.rds

# └── tcga

# └── pheno.rds

***Note:*** Only newly generated files are listed here.

### Transcriptional risk identification

**Timing: 2 h**

To understand the clinical significance of various transcriptional pathways, we correlate transcriptional data to parameters that approximate disease severity. For the cancer datasets, we use mortality data whereas for the atherosclerosis datasets we have to rely on disease severity scores like DUKE and SYNTAX (in the case of STARNET) or on prospective clinical events (BiKE). For ease of reference, we term this transcriptional data correlated to disease severity the “transcriptional risk profile.” These steps are dedicated to identifying the transcriptional risk profile by fitting survival models for cancer TCGA and CVD-events for the BiKE datasets to generate the summary statistics correlating each gene with the disease severity, which includes the effect size and *P*-value. Next, we aggregate the risk profile from gene-level to pathway level.

3. Fit survival models to define the transcriptional risk profile.

> snakemake --cores all Gene

>

> tree results

# results

# └── gene

# ├── AOR_duke.rds

# ├── AOR_syntax.rds

# ├── AOR_vs_MAM.rds

# ├── bike_plaque.rds

# ├── ACC.rds

# └── ……

***Note:*** The downloading, compiling, and model fitting steps are dataset specific. However, the results in the directory “results/gene/” are standardized. Each R Data (RDS) file contains a table that stores the summary statistics (beta and pval), and the Gencode annotations (ensembl, entrez and symbol. The filenames serve as unique identifiers for the corresponding datasets. This standard structure simplifies the process of preparing results for analogous datasets, making it easier for researchers to incorporate them into subsequent analysis steps with minimal modification.

4. Perform a Gene Set Enrichment Analysis (GSEA) on hallmark gene sets to pinpoint the high-risk pathways.

> snakemake --cores all Pathway

>

> tree results/pathway

# results/pathway

# ├── AOR_duke.rds

# ├── AOR_syntax.rds

# ├── AOR_vs_MAM.rds

# ├── bike_plaque.rds

# ├── ACC.rds

# └── ……

### Dataset clustering and identification of shared risk

**Timing: 1 h**

This step enables the detection of subgroups within the list of datasets using clustering analysis, laying the groundwork for identifying the shared risk pathways.

5. Cluster the datasets based on risk pathways.

> snakemake --cores all Cluster

>

> tree -P “∗-clustering.html” reports/

# reports

# ├── athero_and_cancer-clustering.html

# └── cancer_only-clustering.html

***Note:*** The clustering parameters should be fine-tuned in accordance with available knowledge and data.

6. Adjust the dataset grouping configuration in "config.yaml" as needed.

***Note:*** If new subgroups emerge during the clustering, researchers can make adjustments to the configuration file "config.yaml" and include the subgroup definitions for following analyses. For instance, in this example, certain TCGA cancer types will be found to group closely with atherosclerosis and be named as atherosclerosis-similar cancers and the rest as atherosclerosis-dissimilar cancers. When looking exclusively at cancer types, we find that cancers clustered into three main groups, which we name inflammatory, proliferative, and metabolic cancers based on their overarching pathway dysregulation. Once these subgroup definitions are incorporated into the "config.yaml", it is possible to identify shared risk genes and pathways not only between atherosclerosis and cancers, but also between atherosclerosis and atherosclerosis-similar cancers, and so forth.

7. Identify the shared risk genes and pathways.

> snakemake --cores all Common

>

> tree -P “∗-shared_risks.html” reports/

# reports

# ├── athero_vs_cancer-shared_risks.html

# ├── athero_vs_cancer2-shared_risks.html

# └── cancer_clusters-shared_risks.html

### Compound screening and validation

**Timing: several days to weeks**

This step leverages the NIH Library of integrated networks-based cellular signatures (LINCS) drug perturbation database to predict drug/compound efficacy in reversing each dataset’s risk profile. For those highly ranked drugs with sufficient statistical power for validation using electronic health records, this step further executes pharmacovigilance studies to test the drugs’ effectiveness.

8. Screen compounds that could reverse the transcriptional risk profile.

> snakemake --cores all Compound

>

> tree -P “rges.rds” results/compound

# results/compound

# └── rges.rds

***Note:*** This step is computationally taxing and therefore it is highly recommended that this step be executed on a high-performance computing cluster. The output “results/compound/rges.rds” is a table containing the reversal gene expression score (RGES) and associated *P*-value for each compound perturbation assay in each dataset.

9. Prioritize the highly ranked on-market drugs that possess adequate statistical power for testing using electronic health records and design a pharmacovigilance study.

***Note:*** we use Clopidogrel as an example. Clopidogrel is predicted to have specific benefit for certain cancer types. To validate this prediction, we plan to collect two propensity-matched (matched on demographics, smoking status, comorbid conditions, procedures, and therapeutics in the 6 months leading up to enrollment) cohorts. Clopidogrel is generally prescribed to patients diagnosed with cardiovascular events like myocardial infarction. Thus, those indications are defined as the entry events. Patients prescribed clopidogrel will be then in the treatment cohort, while those that are not prescribed this drug will be in the control cohort. The endpoint is defined as the incidence of different cancer types within 5 years.

10. Once the study design is finalized, prepare the configuration files (see examples in EHR-OMOP/conf) by translating the drug, diseases, and indications to OMOP Vocabulary concept IDs, using Athena (https://athena.ohdsi.org/search-terms/terms).
11. Setup the credentials for accessing the EHR database and initiate the pharmacovigilance study.

> snakemake --cores all EHR_validation

>

> tree EHR-OMOP/fit EHR-OMOP/plot

# EHR-OMOP/fit

# ├── clopidogrel-inflammatory.rds

# ├── clopidogrel-metabolic.rds

# ├── clopidogrel-proliferative.rds

# ├── clopidogrel.csv

# └── clopidogrel.rds

# EHR-OMOP/plot

# ├── clopidogrel-inflammatory.pdf

# ├── clopidogrel-metabolic.pdf

# └── clopidogrel-proliferative.pdf

***Note:*** It is highly recommended that this step be executed on a high-performance computing cluster. The output “EHR-OMOP/fit/clopidogrel.csv” is a table containing the survival statistics.

## Expected outcomes

**Figure 1:**
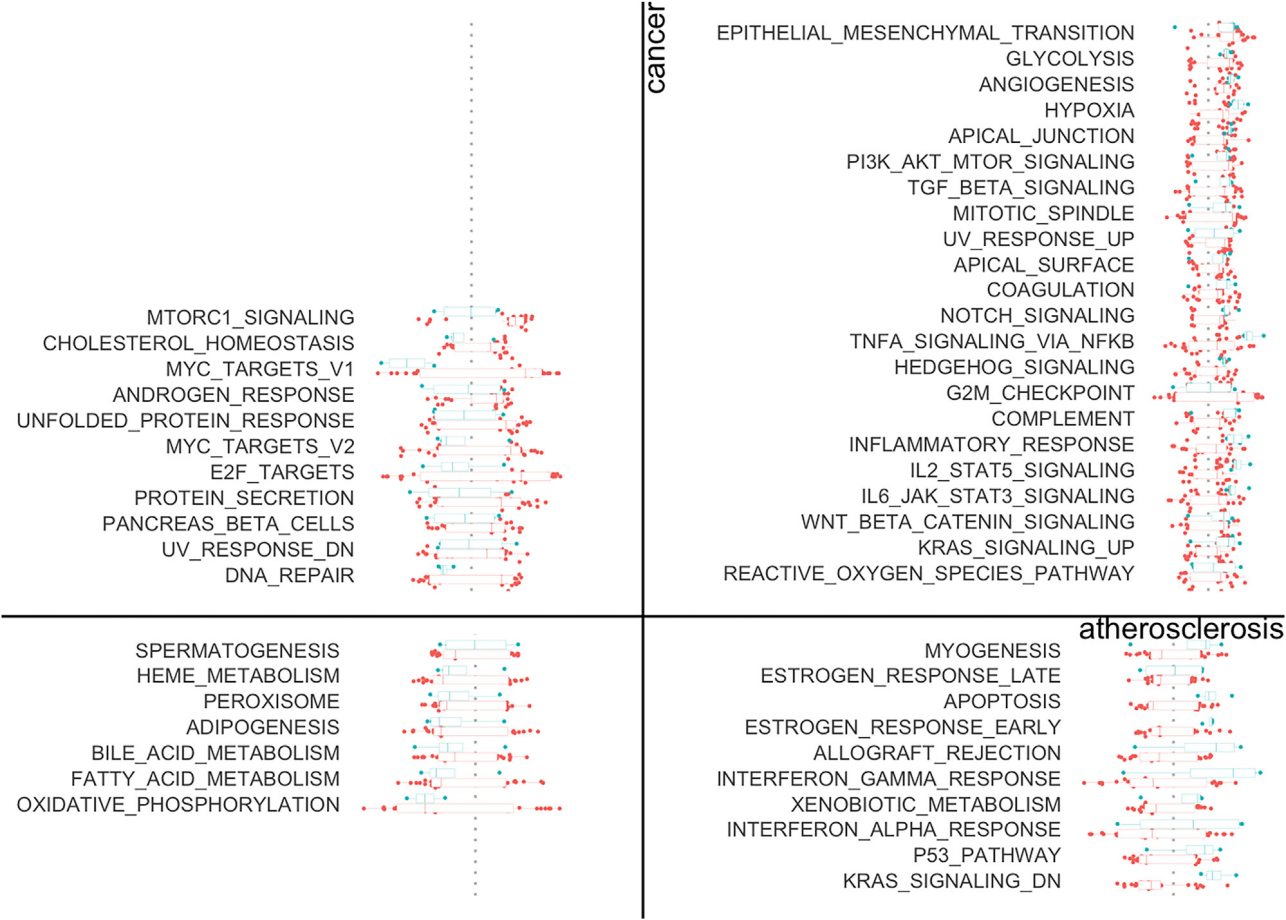
Quadrant plot summarizing the shared transcriptional risks between atherosclerosis and cancer This example plot comes from “reports/athero_vs_cancer-shared_risks.html”. Each boxplot with dots represents the distribution of normalized enrichment score (NES) per pathway for each disease. Blue color denotes atherosclerosis and red denotes cancer. Figure reprinted with permission from Baylis et al., 2023.

A key outcome of this pipeline is a quadrant plot summarizing the shared risks between diseases (step 7). For example, in Figure 1, the pathways in quadrant 1 are common pathways for atherosclerosis and cancer enriched with detrimental genes, while the pathways in quadrant 3 are common pathways enriched with beneficial gene.

**Figure 2:**
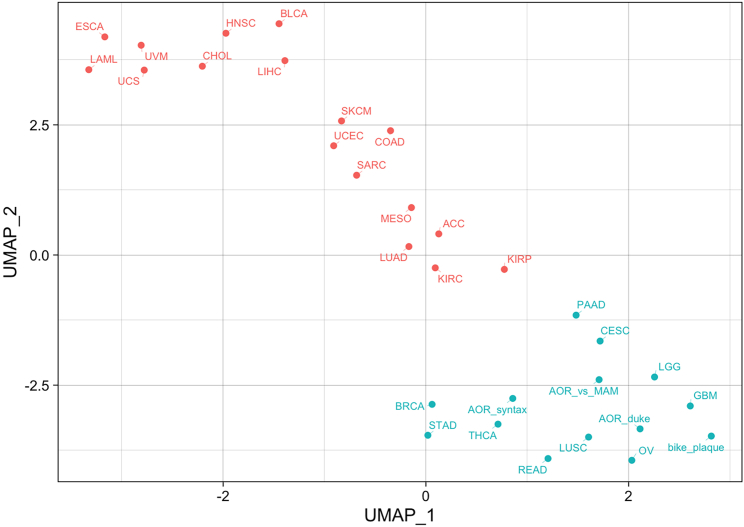
Clustering results for TCGA cancer and CVD datasets This example plot comes from “reports/athero_and_cancer-clustering.html”. Each dot represents a dataset, and the clusters are color-coded. UMAP_1 and UMAP_2 refer to the first two principal components captured by the UMAP (Uniform Manifold Approximation and Projection) algorithm, which facilitates the visualization of high dimensional data. Figure reprinted with permission from Baylis et al., 2023.

Additionally, clustering (step 5) could reveal interesting insights into disease heterogeneity. For example, when clustering atherosclerosis with cancer datasets, certain cancer types grouped more closely with atherosclerosis (Figure 2).

**Figure 3:**
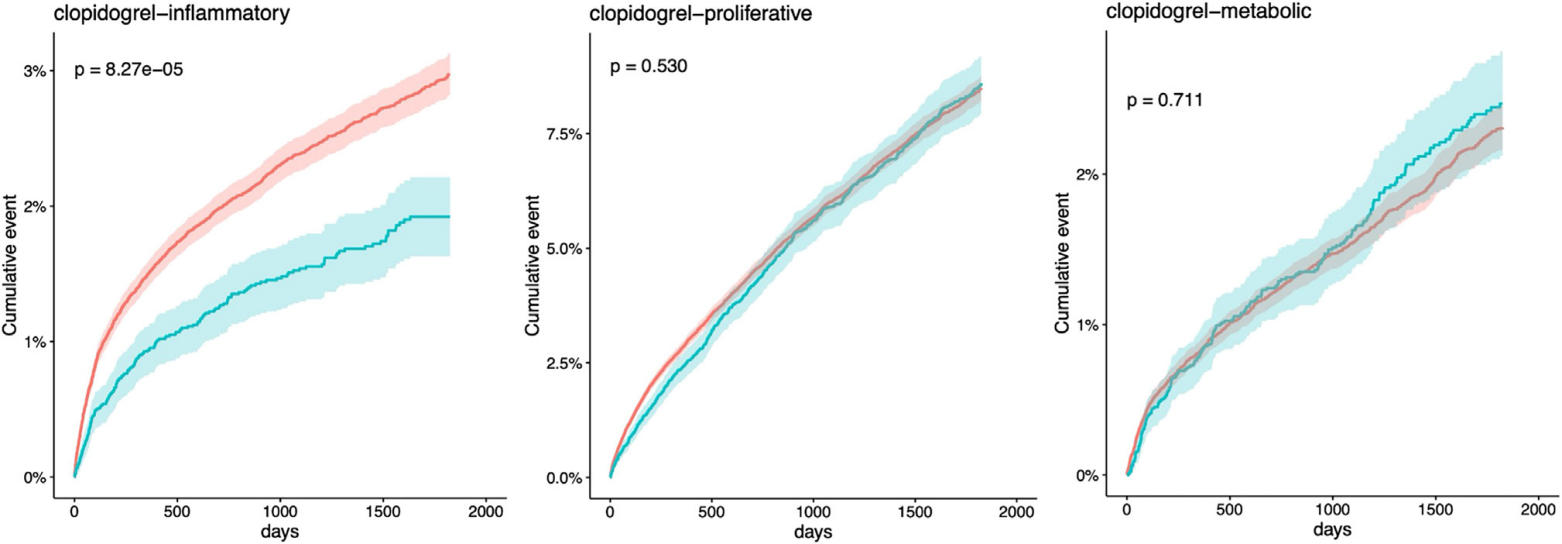
Kaplan-Meier curves of cancer incidence in clopidogrel-treated and control cohorts This example is from “EHR-OMOP/plot/”. The red color denotes the control cohort and green denotes clopidogrel-treated cohort. The endpoints are inflammatory cancers, proliferative cancers, and metabolic cancers, respectively. P value is resulted from the Cox proportional hazards model on the propensity-matched cohort, adjusted for gender, age, race, and smoking history. The shaded band denotes the 95% confidence interval. Figure reprinted with permission from Gao et al., 2022.

This pipeline can also validate drug screening results using electronic health records by collecting two propensity-matched cohorts and fitting survival models to test whether the drug can reduce the incidence of expected diseases (step 11). For example, in Figure 3, Clopidogrel is associated with a reduced incidence of inflammatory cluster cancers as predicted in step 8.

## Limitations

This protocol includes resources for downloading, compiling, and transcriptional risk identification steps, which are tailored specifically to the TCGA datasets, STARNET and BiKE datasets. When working with analogous datasets, researchers may need to prepare the gene level risk statistics file and adjust the configuration file accordingly. Additionally, this protocol leverages the NIH Library of integrated networks-based cellular signatures (LINCS) drug perturbation dataset to screen compounds capable of reversing the transcriptional risk. Notably, since most cell lines in LINCS dataset are cancer cell lines, the screen results are particularly robust and reliable for cancer-related diseases, applying this dataset to other diseases may require approximation (for example using the most abundant cell type or cell type of interest for that disease).

## Troubleshooting

### Problem 1

Some software or packages are unavailable.

### Potential solution

Ensure every step is executed within the designated computing environment, which should be set up correctly as outlined in “Before you begin” step 2–4.

### Problem 2

Some dependent file is unavailable.

### Potential solution

• Ensure the restricted access files are manually downloaded and moved to the correct directory, as outlined in “Before you begin” steps 5–6.
• Ensure every step is executed within the pipeline directory “protocol-cancer-cdv-similarity”.

### Problem 3

Upon executing the command.

> snakemake --cores all Gene

subsequent to custom datasets registration, an error message “Unrecognized file format” is encountered.

### Potential solution

Ensure the custom dataset file adheres to one of the supported formats: Excel (.xls and .xlsx), TSV (.tsv), CSV (.csv), and R Data (.rds). Confirm that the file’s extension accurately reflects its format. An incompatible extension will trigger the error.

### Problem 4

Upon executing the command.

> snakemake --cores all Gene

subsequent to custom datasets registration, an error message “Column `beta` and `pval` are required” or “Column `ensembl` or `entrez` or `symbol` are required” is encountered.

### Potential solution

Ensure the custom dataset file fulfills the following mandatory requirements: at least one gene ID column (**ensembl**, **entrez**, and/or **symbol**), **beta** column, and **pval** column. Missing any of the required columns will trigger the error.

### Problem 5

If you encounter unreasonable pathway enrichment results and consider inspecting the intermediate gene-level risk statistics and pathway-level enrichment metrics for potential insights.

### Potential solution

You can dump the R Data file into an Excel file or TSV file for convenient manual inspection. For example, to inspect the gene-level risk statistics for TCGA BRCA dataset in “results/gene/BRCA.rds”, execute.

> snakemake --cores all results/gene/BRCA.xlsx

and the target Excel file will be generated. You can further inspect whether the gene ID mapping is problematic due to such as unexpected lower case of gene symbol.

## Resource availability

### Lead contact

Further information and requests for resources and reagents should be directed to and will be fulfilled by the lead contact, Nicholas J. Leeper (nleeper@stanford.edu).

### Technical contact

Technical questions on executing this protocol should be directed to and will be answered by the technical contact, Hua Gao (ghbore@gmail.com).

### Materials availability

This study did not generate new unique reagents.

### Data and code availability

The code generated during this study is available at GitHub: https://github.com/ghbore/protocol-cancer-cvd-similarity (https://doi.org/10.5281/zenodo.10493942). All resources and intermediate results bundle are available at Zenodo: https://doi.org/10.5281/zenodo.10032149.
